# A Circulating miRNA-Based Scoring System Established by WGCNA to Predict Colon Cancer

**DOI:** 10.1155/2019/1571045

**Published:** 2019-12-01

**Authors:** Da Qin, Rui Wei, Si Liu, Shengtao Zhu, Shutian Zhang, Li Min

**Affiliations:** Department of Gastroenterology, Beijing Friendship Hospital, Capital Medical University, National Clinical Research Center for Digestive Disease, Beijing Digestive Disease Center, Beijing Key Laboratory for Precancerous Lesion of Digestive Disease, Beijing 100050, China

## Abstract

**Introduction:**

Circulation microRNAs (miRNAs) perform as potential diagnostic biomarkers of many kinds of cancers. This study is aimed at identifying circulation miRNAs as diagnostic biomarkers in colon cancer.

**Methods:**

We conducted a weighted gene coexpression network analysis (WGCNA) in miRNAs to find out the expression pattern among circulation miRNAs by using a “WGCNA” package in R. Correlation analysis was performed to find cancer-related modules. Differentially expressed miRNAs (DEmiRs) in colon cancer were identified by a “limma” package in R. Hub gene analysis was conducted for these DEmiRs in the cancer-related modules by the “closeness” method in cytoscape software. Then, logistic regression was performed to identify the independent risk factors, and a scoring system was constructed based on these independent risk factors. Then, we use data from the GEO database to confirm the reliability of this scoring system.

**Results:**

A total of 9 independent coexpression modules were constructed based on the expression levels of 848 miRNAs by WGCNA. After correlation analysis, green (cor = 0.77, *p* = 3 × 10^‐25^) and yellow (cor = 0.65, *p* = 6 × 10^‐16^) modules were strongly correlated with cancer development. 20 hub genes were found after hub gene analysis in these DEmiRs by cytoscape. Among all these hub genes, hsa-miR-23a-3p (OR = 2.6391, *p* = 6.23 × 10^‐5^) and hsa-miR-663a (OR = 1.4220, *p* = 0.0069) were identified as an independent risk factor of colon cancer by multivariate regression. Furthermore, a scoring system was built to predict the probability of colon cancer based on both of these miRNAs, the area under the curve (AUC) of which was 0.828. Data from GSE106817 and GSE112264 was used to confirm this scoring system. And the AUC of them was 0.980 and 0.917, respectively.

**Conclusion:**

We built a scoring system based on circulation hub miRNAs found by WGCNA to predict the development of colon cancer.

## 1. Introduction

The incidence of colon cancer continues to grow these years. According to large-scale epidemiological studies [1, 2], colon cancer has become a major disease threatening patients' health. Researchers predicted that, in 2019, 145,600 new colon cancer would be diagnosed and 51,020 persons will die because of this disease [1]. Colon cancer develops insidiously, which means that it will not easily exhibit specific symptoms until turning into extremely terminal stages. This will cause irreparable effect to patients. Screening methods which could find colon cancer patients from population were important.

Although guidelines [3, 4] emphasized the importance of noninvasive tools to screen colon cancer, screening of colon cancer still relies on colonoscopies for high-risk population, which would be difficult to popularize in China because of its pain and poor compliance of patients. Moreover, traditional tumor biomarkers ([5], p., 1), such as CEA, were characterized by poor sensitivity and specificity, which may lead to missed diagnosis and waste of medical resources. The establishment of a sensitive and specific serological screening method is particularly important to improve the detection rate of colon cancer.

Previous studies [6–11] have revealed that many kinds of miRNAs play crucial roles in tumorigenesis. miRNAs could be found in many kinds of biofluids, such as blood and urine [12]. So, there would be a potential way to develop circulating miRNAs as minimally invasive biomarkers of cancer.

WGCNA is a method to analyze gene expression patterns in multiple samples. Genes with similar expression patterns would be clustered, and correlation between modules and specific traits or phenotypes could be calculated. Therefore, WGCNA [13] is widely used in the study of cancer researches. In this study, WGCNA-based methods were used to identify gene modules correlated with the development of colon cancer, and the most representative miRNAs were identified as circulation markers to help in screening colon cancer.

## 2. Materials and Methods

### 2.1. Weighted Gene Coexpression Network Analysis

We conducted a weighted gene coexpression network analysis in miRNAs to find out the expression pattern among circulation miRNAs by using a “WGCNA” package [14] in R. Data was from GSE61741 [15] in Gene Expression Omnibus (GEO) database. miRNAs with a similar expression pattern were regarded as a specific module and marked by a unique color. Correlations between these modules were calculated, and heat map was performed to show the independence between each of these modules. Then, correlation analysis was performed to find cancer-related modules. Modules which are strongly correlated with cancer development were chosen to undergo further analysis.

### 2.2. Hub Gene Selection

Differentially expressed miRNAs in colon cancer were identified by a “limma” [16] package in R. Hub gene analysis was conducted for these DEmiRs in the cancer-related modules by the closeness method in cytoscape [17] software. miRNA-miRNA interaction graph was performed to visualize the relationship between these circulation miRNAs.

### 2.3. Scoring System Establishing

Logistic regression was performed to identify the independent risk factor, and the scoring system was constructed based on the independent risk factors. Furthermore, we visualized this scoring system by nomogram using an “rms” package in R. A receiver operating characteristic (ROC) curve was performed to evaluate the reliability of this scoring system using a “pROC” package [18] in R.

### 2.4. External Validation

To confirm the reliability of this scoring system, data from GSE106817 [19] and GSE112264 [20] database was used. Violin graphs were performed to describe gene expression levels. ROC curves were graphed to evaluate the reliability of this scoring system using a “pROC” package [18] in R.

### 2.5. Statistical Analysis

All statistical analyses were performed using R software (version 3.5.2; https://www.r-project.org/). WGCNA were conducted by a “WGCNA” package (version 1.68). Differentially expressed miRNAs were identified by a “limma” package (version 3.38.3). Then, hub gene analysis was conducted by the closeness method in cytoscape software (version 3.7.1). And an “rms” package (version 5.1-3.1) was used to visualize the scoring system. Then, ROC curves were graphed using a “pROC” package (version 1.15.3). Statistical significance was defined as *p* < 0.05.

## 3. Results

### 3.1. Weighted Gene Coexpression Network Analysis

**Figure 1:**
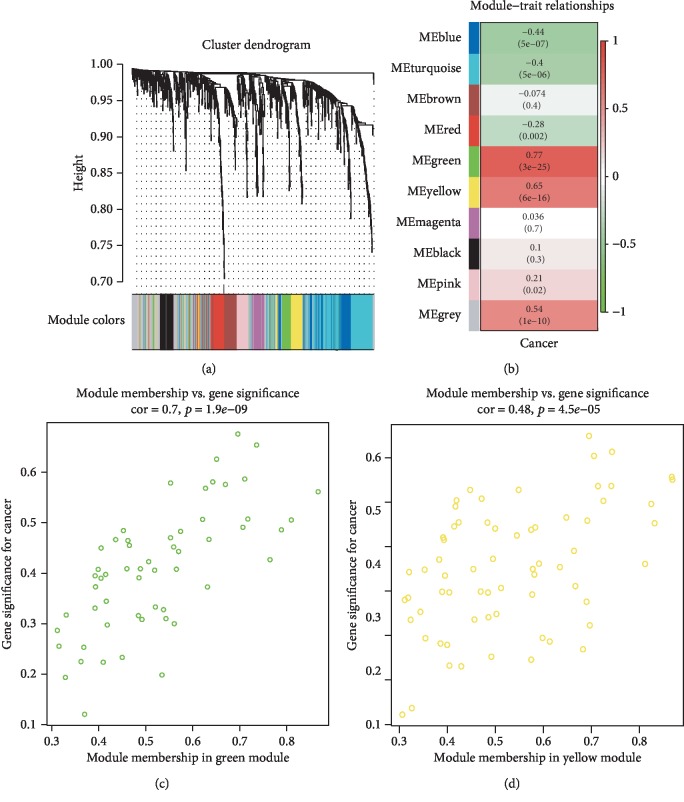
Weighted gene coexpression network analysis. (a) Gene coexpression module construction. (b) Correlation analysis between gene modules and cancer development. (c, d) Scatter diagrams between each gene in modules and cancer development.

123 samples from GSE61741 were clustered based on their gene expression patterns (Supplementary [Supplementary-material supplementary-material-1]). Then, a total of 9 coexpression modules were constructed based on the expression levels of 848 miRNAs by WGCNA (Figure 1(a)). Heat maps were performed to prove the independence between each gene (Supplementary [Supplementary-material supplementary-material-1]). After correlation analysis, green (cor = 0.77, *p* = 3 × 10^‐25^) and yellow (cor = 0.65, *p* = 6 × 10^‐16^) modules were strongly correlated with cancer development (Figure 1(b), Supplementary [Supplementary-material supplementary-material-1]). Scatter diagrams were graphed to prove the correlation between each gene in modules and cancer development (Figures 1(c) and 1(d)).

### 3.2. Hub Gene Selection

**Figure 2:**
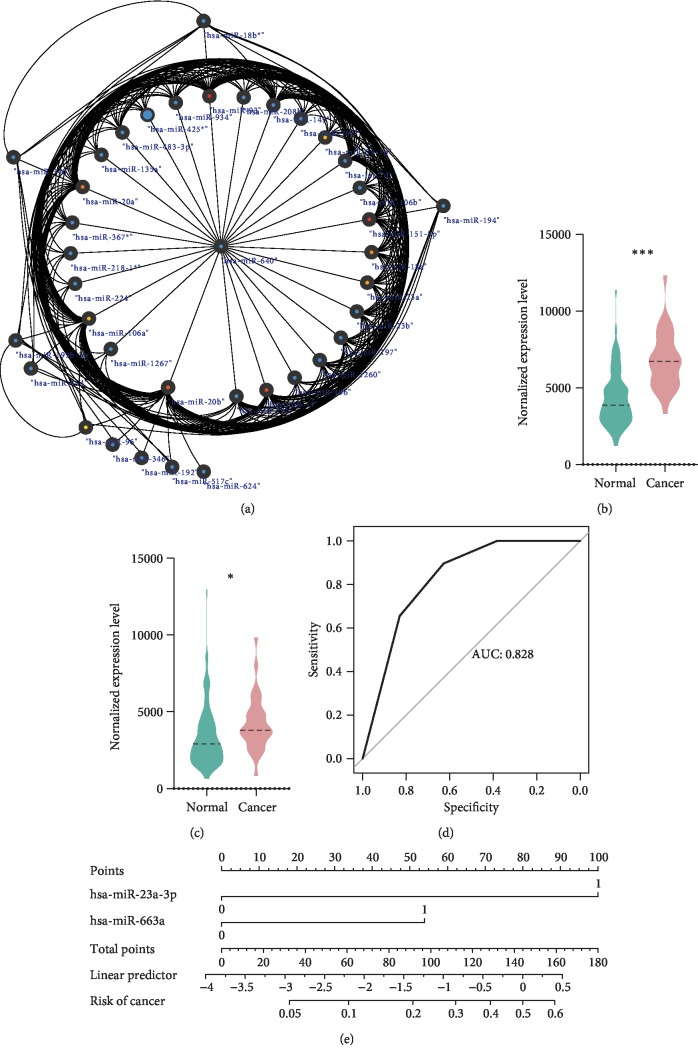
Hub gene selection and scoring system establishment. (a) miRNA-miRNA interaction network in green module. (b) hsa-miR-23a-3p levels were significantly increased in the blood of colon cancer. (c) hsa-miR-663a levels were significantly increased in the blood of colon cancer. (d) ROC of a scoring system. (e) Nomogram of a scoring system.

DEmiRs in these modules were found by a “limma” package, and after hub gene analysis in these DEmiRs by closeness software in cytoscape, 20 hub genes were identified (Table 1). The miRNA-miRNA interaction network is shown in Figure 2(a) and Supplementary [Supplementary-material supplementary-material-1].

### 3.3. Scoring System Establishing

hsa-miR-23a-3p (Figure 2(b)) and hsa-miR-663a (Figure 2(c)) levels were significantly increased in the blood of colon cancer, *p* values of which were <0.0001 and 0.0388, respectively. Then, hsa-miR-23a-3p (OR = 2.6391, *p* = 6.23 × 10^−5^) and hsa-miR-663a (OR = 1.4220, *p* = 0.0069) were identified as an independent risk factor of colon cancer by multivariate regression. Furthermore, a scoring system was built to predict the probability of colon cancer based on both of these miRNAs. The area under the curve (AUC) of this scoring system was 0.828 (Figure 2(d)). A nomogram was performed to visualize the scoring system (Figure 2(e)).

### 3.4. External Validation

Data from GSE112264 and GSE106817 was used to confirm this scoring system.

**Figure 3:**
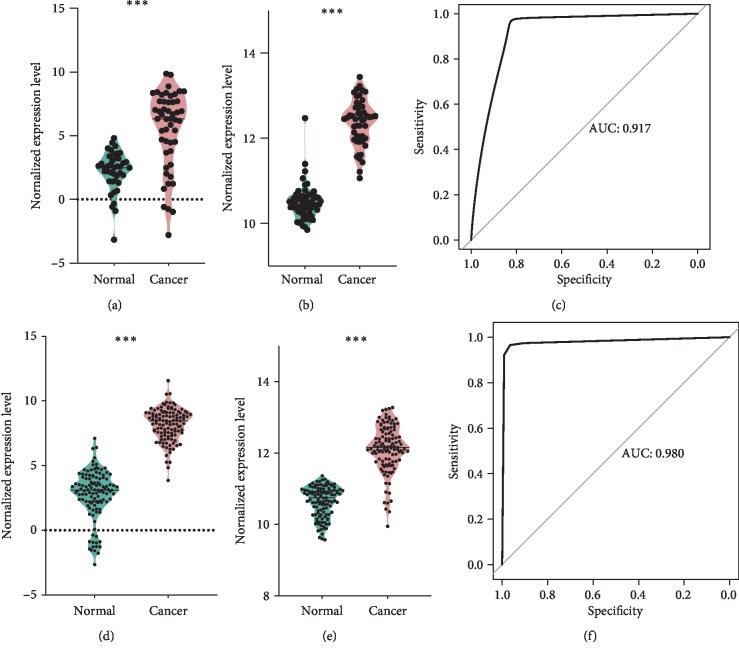
External validation. (a) Colon cancer patients have higher hsa-miR-23a-3p levels in GSE112264. (b) Colon cancer patients have higher hsa-miR-663a levels in GSE112264. (c) ROC of a scoring system in GSE112264. (d) Colon cancer patients have higher hsa-miR-23a-3p levels in GSE106817. (e) Colon cancer patients have higher hsa-miR-663a levels in GSE106817. (f) ROC of a scoring system in GSE106817.

For GSE112264, a total of 41 blood samples of healthy control and 50 blood samples of colon cancer were analyzed. hsa-miR-23a-3p (Figure 3(a)) and hsa-miR-663a (Figure 3(b)) levels were significantly increased in the blood of colon cancer, *p* values of which were both <0.0001. The receiver operating curve is shown as Figure 3(c), AUC of which was 0.917. When choosing a threshold of 0.7, the sensitivity of this scoring system was 0.97 and the specificity was 0.82.

For GSE106817, a total of 115 blood samples of healthy control and 115 blood samples of colon cancer were analyzed. hsa-miR-23a-3p (Figure 3(d)) and hsa-miR-663a (Figure 3(e)) levels were significantly increased in the blood of colon cancer, *p* values of which were both <0.0001. ROC is shown as Figure 3(f), AUC of which was 0.980. When choosing a threshold of 0.5, the sensitivity of this scoring system was 0.965 and the specificity was 0.965.

## 4. Discussion

Colon cancer develops insidiously. Screening methods which could find colon cancer patients from population were important. Although guidelines [3] emphasized the importance of noninvasive tools, such as the fecal immunochemical test [21–24], high-sensitivity guaiac-based fecal occult blood test [25, 26], and computed tomography colonography, to screen colon cancer, screening of colon cancer in China still relies on colonoscopies for high-risk population. A new kind of noninvasive way to diagnose CC is urgently needed.

Circulation miRNAs have been regarded as an effective noninvasive screen method for many kinds of cancers [27–29]; some studies about circulation miRNAs have been performed in colon cancer. It has been proved that circulation miRNAs could hold stable under various storage conditions [30], which provided basic theoretical support for miRNAs to become tumor biomarkers.

Several researchers did great jobs in identifying a miRNA biomarker for colon cancer. Sabry et al. [31] focused on HIF-1*α*-VEGF signaling pathway-related miRNAs, such as miR-210 and miR-21. They found that miR-210 and miR-21 were associated with clinical staging of colon cancer and showing a high diagnostic value with sensitivity and specificity 88.6%, 90.1% and 91.4%, 95.0%, respectively. Bilegsaikhan et al. [32] studied miR-338-5p which has been reported to be upregulated in colon cancer tissues and found that the AUC of it was 0.923 in distinguishing CRC from healthy controls. Moreover, Zhang et al. [33] studied the miRNA profile in the serum of patients with colon cancer by microarray. And they identified 8 DE miRNAs including miR-4463, miR-5704, miR-371b-3p, miR-1247-5p, miR-1293, miR-548at-5p, miR-107, and miR-139-3p, to distinguish colon cancer patients from normal controls. However, no diagnostic tests and AUC analysis were performed in this study. Krawczyk et al. [34] focused on the combination of circulation miR-506 and miR-4316, which showed an AUC value of 0.751 to predict colon cancer.

Their findings were mostly based on the aberrantly expressed circulation miRNAs or miRNA which has been reported upregulating in colon cancer tissues; deep data mining and analysis were lacking. Our study was based on both differentially expressed miRNAs and WGCNA. Instead of only focusing on differentially expressed miRNAs like most of the other researches, WGCNA utilizes information from all of the most varied genes, to identify interested gene module and to analyze their association with phenotypes. By making full usage of gene expression information, we can find hub genes which really matter in the development of cancers. Hub genes we discovered might not have the smallest *p* value in DE analysis; however, it would be more stable and more biologically meaningful than genes selected by *p* values of DE analysis, which could be proved by our validation datasets. This article chose a more powerful dimension to select target genes, which would explain why miR-23 and miR-663 were not selected in the other studies.

We have identified hsa-miR-23a-3p (OR = 2.6391, *p* = 6.23 × 10^−5^) and hsa-miR-663a (OR = 1.4220, *p* = 0.0069) as diagnostic biomarkers in colon cancer using data from GSE61741 and confirmed our scoring system in GSE112264 and GSE106817. The AUC of these two databases was 0.917 and 0.980, respectively. Diagnostic test results reflect that it is reliable and stable for our scoring system to distinguish cancer patients from population, which means that it is possible to regard hsa-miR-23a-3p and hsa-miR-663a as diagnostic biomarkers in colon cancer.

For hsa-miR-663a, Kuroda et al. [35] indicated that after treatment with antimicrobial peptides, miR-663a could regulate cancer development of CC by targeting the CXCR4-p21 pathway. And for hsa-miR-23a-3p, no previous researches were performed to find its function in colon cancer. In hepatocellular cancer (HCC), Liu et al. [36] found that HCC patients with a high body fat ratio have higher miR-23a levels in both serum exosomes and tumor tissues than HCC patients with a low body fat ratio. Their further studies indicated that miR-23a was transported into cancer cells via exosomes, thus promoting HCC cell growth and migration. Our results exhibited that miR-23a was upregulated stably in blood samples of colon cancer patients, and it was also one of the hub genes in cancer-related modules, which indicated that hsa-miR-23a-3p could exhibit similar properties as HCC in colon cancer.

This is the first WGCNA research to identify circulation miRNA in colon cancer. The diagnostic biomarkers we defined were stable and reliable. Our study was based on the GEO database; although advanced analysis methods and multidatabase validation were applied, it still has some limitations because no hospital's samples were involved. The prospective validation using our hospital's samples would be performed when our cohorts are strong enough to validate these findings. Besides, we found that two hub miRNAs could be used as a circulation biomarker for colon cancer; however, no functional mining experiments were performed. Our further studies would focus on the function of hsa-miR-23a-3p and hsa-miR-663a in colon cancer tissues and circulation system to find its roles in CC development.

## 5. Conclusion

In this study, we built a scoring system based on circulation hub miRNAs found by WGCNA to predict the development of colon cancer.

## Figures and Tables

**Table 1:** Hub genes identified in each module.

miRNA name	Score
Green module	
hsa-miR-93	5.41
hsa-miR-151-3p	5.16
hsa-miR-17	5.03
hsa-miR-20b	5.00
hsa-miR-20a	4.23
hsa-miR-18a	3.70
hsa-miR-23a	3.56
hsa-miR-28-3p	3.48
hsa-miR-106a	3.48
hsa-miR-96	3.15
Yellow module	
hsa-miR-1915	8.40
hsa-miR-92b^∗^	7.73
hsa-miR-658	7.40
hsa-miR-1908	7.36
hsa-miR-675	7.25
hsa-miR-663	7.05
hsa-miR-135a^∗^	6.31
hsa-miR-1275	5.85
hsa-miR-885-3p	5.65
hsa-miR-374a	5.65

## Data Availability

All the data used in this article could be downloaded in the GEO database.

## Supplementary Materials

Supplementary Materials Supplementary Figure A: sample clustering. Supplementary Figure B: heat map proving the independence between each gene. Supplementary Figure C: correlation analysis between gene modules and cancer development. Supplementary Figure D: miRNA350-miRNA interaction network in yellow module.
