# Supplementary figures and images for: A Circulating miRNA-Based Scoring System Established by WGCNA to Predict Colon Cancer
Source: Anal Cell Pathol (Amst). 2019 Dec 1;2019:1571045. doi: 10.1155/2019/1571045 (PMC6913280; doi:10.1155/2019/1571045)

## 2

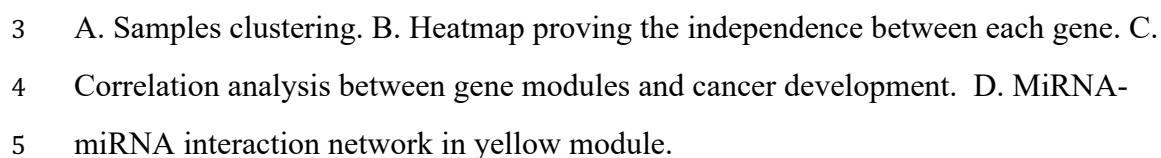

Supplement: Supplementary Materials — Supplementary Figure A: sample clustering. Supplementary Figure B: heat map proving the independence between each gene. Supplementary Figure C: correlation analysis between gene modules and cancer development. Supplementary Figure D: miRNA350-miRNA interaction network in yellow module. [file 1571045.f1.pdf]
